# Robot Path Planning Based on Genetic Algorithm Fused with Continuous Bezier Optimization

**DOI:** 10.1155/2020/9813040

**Published:** 2020-02-25

**Authors:** Jianwei Ma, Yang Liu, Shaofei Zang, Lin Wang

**Affiliations:** School of Information Engineering, Henan University of Science and Technology, Luoyang 471023, Henan, China

## Abstract

In this study, a new method of smooth path planning is proposed based on Bezier curves and is applied to solve the problem of redundant nodes and peak inflection points in the path planning process of traditional algorithms. First, genetic operations are used to obtain the control points of the Bezier curve. Second, a shorter path is selected by an optimization criterion that the length of the Bezier curve is determined by the control points. Finally, a safe distance and adaptive penalty factor are introduced into the fitness function to ensure the safety of the walking process of the robot. Numerous experiments are implemented in two different environments and compared with the existing methods. It is proved that the proposed method is more effective to generate a shorter, smoother, and safer path compared with traditional approaches.

## 1. Introduction

Path planning is an important research direction in the field of mobile robots, and it is one of the main difficulties in research on such robots [1]. The path planning problem aims to find the safest and shortest path autonomously without collisions from the start point to the target point under a given environment with barriers [2, 3]. Path planning has been widely used in fields such as logistics distribution, intelligent transportation, and weapons navigation [4–6]. Therefore, how to find a fast and effective path has become a research issue with a high theoretical significance and practical value.

In recent years, the genetic algorithm (GA) has been widely applied in mobile robot path planning problems because of its great global optimization ability and implicit parallel computing characteristics [7, 8]. The GA searches for the optimal solution by simulating the natural evolution based on the theoretical models of the genetic inheritance and variation in Darwin's biological evolution [9, 10]. Recently, some meaningful results have been reported for the GA. For example, a generalized segmentation crossover operator was introduced into the GA to improve the local optimization ability and execution efficiency of the algorithm [11]. Albayrak and Allahverdi introduced a greedy search algorithm into the GA mutation operation and designed a new greedy sunbath mutation operator to solve the traveling salesman problem (TSP) [12, 13]. Furthermore, an improved crossover operator was proposed [14], in which premature convergence could be avoided to obtain an optimal path in static environments. A robot path planning method was proposed based on the improved genetic algorithm [15], in which the adaptability of a mobile robot path planning algorithm was improved by introducing chromosomes with variable lengths. A parallel elite genetic algorithm was proposed to maintain population diversity, avoid premature convergence, and maintain parallelism with traditional genetic algorithms [16]. However, it is noteworthy that there still exist many problems in the planning process. For example, the spikes and inflection points in the obtained path will make the robot unable to walk along the planned path during the moving process, or it will switch frequently between different modes such as stop, rotate, and restart, which leads to excessive loss of time and energy, etc. [6, 17].

Recently, the Bezier curve has been applied in smooth path planning (see [6, 18–27]). As one example, a genetic algorithm was proposed to find the control points of the segmented Bezier curves and thus solve the problem of the mobile robot path planning [16]. A path planning method was proposed based on the Bezier curve to solve the traveling path in multiagent robot soccer [21]. A collision-free curvature bounded smooth path planning technique was proposed to divide the nodes in the piecewise linear path into control points [22]. Furthermore, a Bezier-curve-based improved genetic algorithm (MGA) was proposed and used for path planning in dynamic fields [23]. A new chaotic particle swarm optimization algorithm (CPSO) was proposed to optimize the control points of the Bezier curve, in which the total distance between the start point and the end point was minimized by using the selected control points [24]. Additionally, a Bezier-curve-based path planner was proposed and applied in autonomous vehicles [25]. Moreover, the collaborative collision avoidance method was presented for multi-incomplete robots based on Bernstein–Bezier curves [26]. Finally, an effective and analytical continuous curvature path smoothing algorithm was proposed [27], and it was found to be suitable for the sequence of path points generated by an obstacle avoidance path planner.

Nonetheless, there is still much difficulty in planning a smooth and safe path for a mobile robot by using the methods mentioned above. Thus, this paper aims to propose a new smooth path planning method to deal with the redundant nodes and peak inflection points in the path planning process. First, genetic operators are used to obtain the control points of the Bezier curve, which ensures the smooth continuity of the path. Second, the length of the Bezier curve is selected to be the optimization criterion to ensure the shortest length of the planned path. Third, by increasing the safety distance and the adaptive penalty factor in the fitness function, the safety of the robot walking process is guaranteed. Finally, experimental results illustrate that the proposed method can produce a shorter, smoother, and safer effective path than the existing methods (e.g., [28–31]). In conclusion, the contributions of this paper are as follows: (1) compared with the methods introduced in [29, 30], our genetic operations are used to obtain the control points of the Bezier curve, which can guarantee the continuity of path curvature; (2) a shorter smooth path is selected by using an optimization criterion, which can ensure that the generated path is optimal without requiring further smoothing; and (3) in contrast with the methods in [6, 22], the safety in robots' walking progress is further improved by introducing an adaptive adjustment in the fitness function.

The rest of the article is organized as follows. The Bezier curve is introduced in Section 2.  Section 3 presents the key points of the smooth path planning method based on the Bezier curve. The experimental results on the feasibility and effectiveness of the proposed method are discussed in Section 4. Finally, a conclusion and directions for future work are presented in Section 5.

## 2. Bezier Curve

The Bezier curve is a continuous smooth curve that is determined by a few feature controls points, a start point, and an end point. The high-order derivative continuity of the Bezier curve ensures the smooth variation of the curve from the start point to the end point [17, 32–34]. Therefore, when the Bezier curve is used to obtain the traveling path of the robot, the planned path is continuous and smooth.

Given a curve with *m* control points, *P*_0_, *P*_1_, *P*_2_,…, *P*_*m*_, the corresponding Bezier curve is determined as in [35]:

(1) $$\begin{array}{c} P\left(t\right)=\sum_{i=0}^{m}B_{i}^{m}\left(t\right)P_{i},\;0\leq t\leq 1, \end{array}$$

(2) $$\begin{array}{c} B_{i}^{m}\left(t\right)=\left(\begin{array}{c} m\\ i \end{array}\right)t^{i}{\left(1-t\right)}^{m-i},\;i=0,1,\ldots ,m, \end{array}$$

where *t* is the positional parameter (e.g., when *t*=0.25, *P*(*t*) is a quarter of the path from point *P*_0_ to *P*_*m*_), *P*_*i*_ represents the control point of the Bezier curve, and *B*_*i*_^*m*^(*t*) is the Bernstein polynomial, which is the basic function of Bezier curve expressions.

According to equations (1) and (2), the derivatives of the Bezier curve can be also determined by the control points. The first derivative of a Bezier curve is expressed as follows:

(3) $$\begin{array}{c} \dot{P}\left(t\right)=\frac{\text{d}P\left(t\right)}{\text{d}t}=m\sum_{i=0}^{m-1}B_{i}^{m-1}\left(t\right)\left(P_{i+1}-P_{i}\right), \end{array}$$

and the higher-order derivatives of the Bezier curve can be calculated by equation (3). The second derivative of the Bezier curve can be expressed as in [35]:

(4) $$\begin{array}{c} \ddot{P}\left(t\right)=m\left(m-1\right)\sum_{i=0}^{m-2}B_{i}^{m-2}\left(t\right)\left({\dot{P}}_{i+2}-2{\dot{P}}_{i+1}+{\dot{P}}_{i}\right). \end{array}$$

Therefore, in the two-dimensional plane, the curvature of the Bezier curve with respect to *t* can be represented as

(5) $$\begin{array}{c} k\left(t\right)=\frac{1}{R\left(t\right)}=\frac{{\dot{P}}_{x}\left(t\right){\ddot{P}}_{y}\left(t\right)-{\dot{P}}_{y}\left(t\right){\ddot{P}}_{x}\left(t\right)}{{\left({\dot{P}}_{x}^{2}\left(t\right)+{\dot{P}}_{y}^{2}\left(t\right)\right)}^{3/2}}, \end{array}$$

where *R*(*t*) stands for the radius of curvature and ${\dot{P}}_{x}\left(t\right)$, ${\dot{P}}_{y}\left(t\right)$, ${\ddot{P}}_{x}\left(t\right)$, and ${\ddot{P}}_{y}\left(t\right)$ are the components of the first and second derivatives of the Bezier curve *P*(*t*) for the *X* and *Y* coordinates.

**Figure 1:**
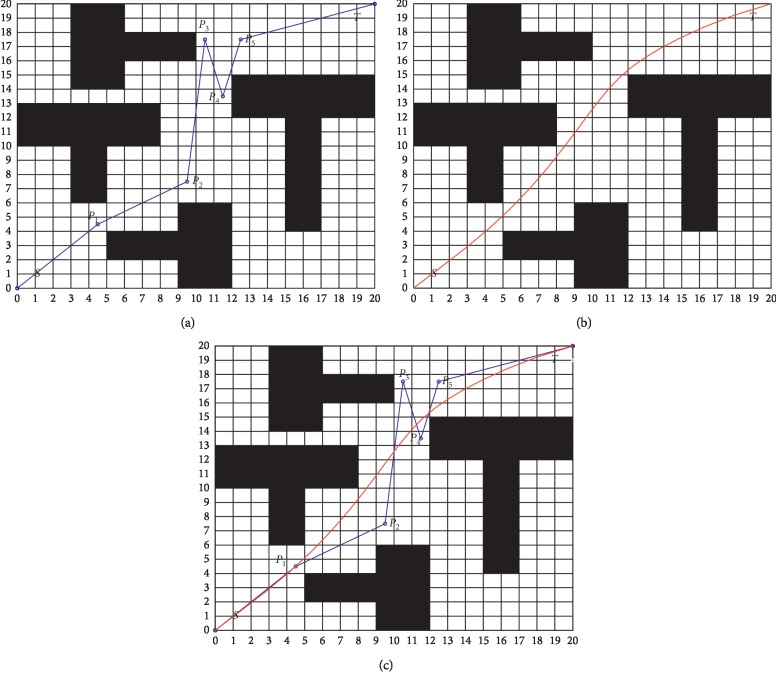
Path planning. (a) Traditional path planning; (b) Bezier curve path planning; (c) comparison of path planning methods.

In general, the traditional algorithm of path planning will produce a lot of sharp inflection points and redundant nodes, as shown in Figure 1(a), where *S* is the start point, *T* is the end point, and *P*_1_, *P*_2_, *P*_3_, *P*_4_, *P*_5_ are the path inflection points. Assuming that *P*_1_, *P*_2_, *P*_3_, *P*_4_, *P*_5_ are the control points, the corresponding Bezier curve can be obtained from equations (1) and (2) (see Figure 1(b)). From Figure 1(c), it can be seen that the path obtained by the Bezier curve is smoother and shorter.

## 3. Smooth Path Planning Based on the Bezier Curve

In this study, the genetic operator and Bezier curve are combined to find a smooth and safe path for the mobile robot. First, the genetic operator is applied to search for the control points of the Bezier curve; then, the safety distance and the adaptive adjustment factor are added to the fitness function to evaluate the safety of the planned Bezier path. Last, the shortest path is determined according to the length of all the planned Bezier paths. The key points of the proposed methods are described below.

### 3.1. Problem Description

The path planning problem is generally described as searching for a collision-free path from the start point to the target point according to certain evaluation indicators. Specifically, the whole planned path from the start point to the end point must be the shortest and collision-free. The problem can be mathematically defined as follows:

(6) $$\begin{array}{c} \text{distance}={\left|P\left(t\right)\right|}_{\min},\;0\leq t\leq 1, \end{array}$$

(7) $$\begin{array}{c} P\left(t\right)\in S⟶T, \end{array}$$

(8) $$\begin{array}{c} P\left(t\right)\in \text{collision}-\text{free,} \end{array}$$

where *t* is the positional parameter, |*P*(*t*)|_min_ stands for the length of the Bezier curve path, *S*⟶*T* represents the path from start point *S* to end point *T*, and collision − free is the path without collision.

Equations (1) and (2) show that the Bezier curve is determined by the control points. Therefore, the problem is equal to finding the control points of the Bezier curve with constraint (9).

(9) $$\begin{array}{c} P_{i}\notin \text{obs}. \end{array}$$

Here, obs is the obstacle, and *P*_*i*_ represents the control points of the Bezier curve. It must hold that the control points cannot be in the obstacle when searching for the control points of the Bezier curve.

### 3.2. Chromosome Encoding

The chromosome needs to be encoded before genetic manipulation (i.e., the chromosome is the control point sequence of the Bezier curve). Common encoding methods include binary encoding and decimal encoding. Binary coding uses strings of 0 or 1 to form a chromosome, which has the advantages of simple operation and easy decoding. Therefore, the chromosome of this paper is encoded in binary.

All control points are defined at the center of the grid in the workspace. The transformation from grid numbers to coordinate values is expressed as

(10) $$\begin{array}{c} P_{x}\left(t\right)=\left\{\begin{array}{c} \mathrm{mod}\left(\text{num},M\right)-0.5,\;\\ M-0.5,\text{if}P_{x}\left(t\right)=-0.5, \end{array}\right. \end{array}$$

(11) $$\begin{array}{c} P_{y}\left(t\right)=M+\text{ceil}\left(\text{num},M\right)+0.5, \end{array}$$

where num indicates the grid number, *M* denotes the size of map, mod stands for the remainder operation, ceil represents rounding down, and *P*_*x*_(*t*) and *P*_*y*_(*t*) are the *X* and *Y* coordinate components of the center of the grid, respectively.

### 3.3. Adaptive Adjustment of Fitness Function

Although the shortest path can be guaranteed when the path length is regarded as the main criterion, there may exist some problems of collision caused by the small distance between the robot and obstacles. Therefore, this chapter proposes a safety-assurance-based fitness function to increase the safety distance by introducing an adaptive penalty factor, which is expressed as follows:

(12) $$\begin{array}{c} \mathrm{fi}t_{\text{new}}=\left\{\begin{array}{cc} {\text{fit}}_{1}, & \text{for feasible paths,}\\ \frac{1}{w_{1}^{*}\mathrm{fi}t_{1}+w_{2}^{*}\mathrm{fi}t_{2}}, & \text{for infeasible paths,} \end{array}\right. \end{array}$$

(13) $$\begin{array}{c} \mathrm{fi}t_{1}=\left|P\left(t\right)\right|, \end{array}$$

(14) $$\begin{array}{c} \mathrm{fi}t_{2}=\left\{\begin{array}{cc} 0, & L_{\text{min}}>L_{e},\\ {\left(1-\frac{L_{\min}}{L_{e}}\right)}^{2}, & L_{\min}<L_{e}, \end{array}\right. \end{array}$$

where *P*(*t*) is the length of the Bezier curve path obtained by the control points sequence under constraints (6)–(9). *L*_*e*_ is the safety distance from the obstacle. When the minimum distance *L*_min_ between the path and the obstacle is less than the safety distance, the penalty will be imposed. Equation (14) shows that the intensity of punishment is related to *L*_min_. The closer the distance between the path and the obstacle, the higher the penalty strength. This enables dynamic adjustment of the fitness function to improve the quality of the planning path. According to equation (12), we can observe that the shorter the planning path and the higher the fitness value of the path, the greater the probability that the path will be selected.

### 3.4. Genetic Operation

In this study, genetic operations are introduced into the Bezier curve to find the control points. There are three genetic operators: the selection operator, crossover operator, and mutation operator.

The selection operator implements the selection of the path by utilizing different selection strategies according to the fitness value. Selection methods are the roulette method, championship method, etc. This paper adopts the roulette method to select the path. Assuming that the path length of the *i*th Bezier curve is |*P*_*i*_(*t*)|, the fitness value of the Bezier curve is fit_*i*_ (see equation (12)). The probability of the selected path is

(15) $$\begin{array}{c} p_{i}=\frac{\mathrm{fi}t_{i}}{\sum_{i=1}^{n}\mathrm{fi}t_{i}}, \end{array}$$

where *n* is the number of Bezier curves.

**Figure 2:**
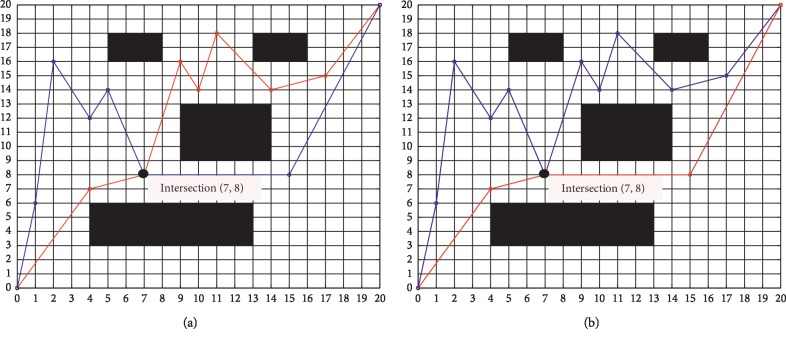
Single-point crossover operation, where intersection (7, 8) is a crossover point. (a) Control points before crossing; (b) control points after crossing.

The crossover operation combines the characteristics of two parent chromosomes to produce two offspring. According to the crossover probability, the genes of two chromosomes are swapped at a randomly generated crossover point. A single-point crossover is adopted in this paper, as is shown in Figure 2.

The mutation operator is introduced to increase the diversity of solutions. It changes the part of the gene by mutating any gene excluding the start point and the end point in the chromosome, and it avoids premature convergence caused by the algorithm falling into local optimum in the solution process. In this paper, the probability of the mutation operator is set to 0.1, which can guarantee the stability of the algorithm's solution process.

## 4. Experiment and Analysis

**Figure 3:**
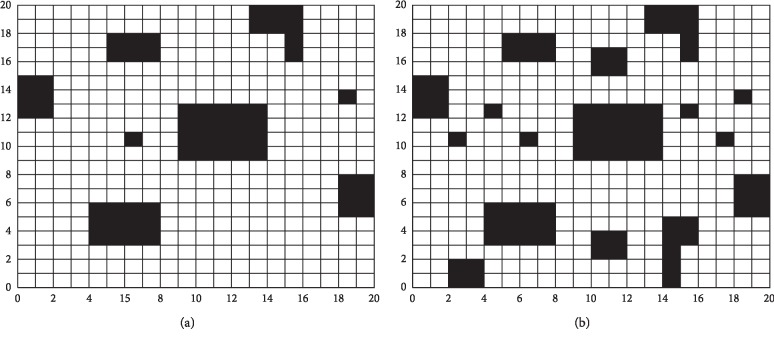
Path planning environments: (a) environment 1; (b) environment 2.

In this section, to verify the feasibility and effectiveness of the proposed algorithm, the path planning of the mobile robot is validated in two different grid environments, as shown in Figure 3. In environment 1, the starting coordinate of the robot is (0,0), the end point coordinate is (20, 20), the obstacle coverage rate is 15.00%, and the parameters are set as follows: the population size is taken as *Q*=100, the maximum generation is taken as gn=50, the crossover probability is taken as Re=0.9, and the mutation probability is taken as Mu=0.1, *w*_1_=0.8, *w*_2_=0.1, and *L*_*e*_=0.25. In environment 2, the starting coordinate of the robot is (0,0), the end point coordinate is (20,20), the obstacle coverage rate is 20.75%, and the parameters are set as follows: the population size is taken as *Q*=100, the maximum generation is taken as gn=100, the crossover probability is taken as Re=0.9, and the mutation probability is taken as Mu=0.1, *w*_1_=0.9, *w*_2_=0.1, and *L*_*e*_=0.25.

### 4.1. Feasibility Experiment of Bezier Smoothing Algorithms

**Figure 4:**
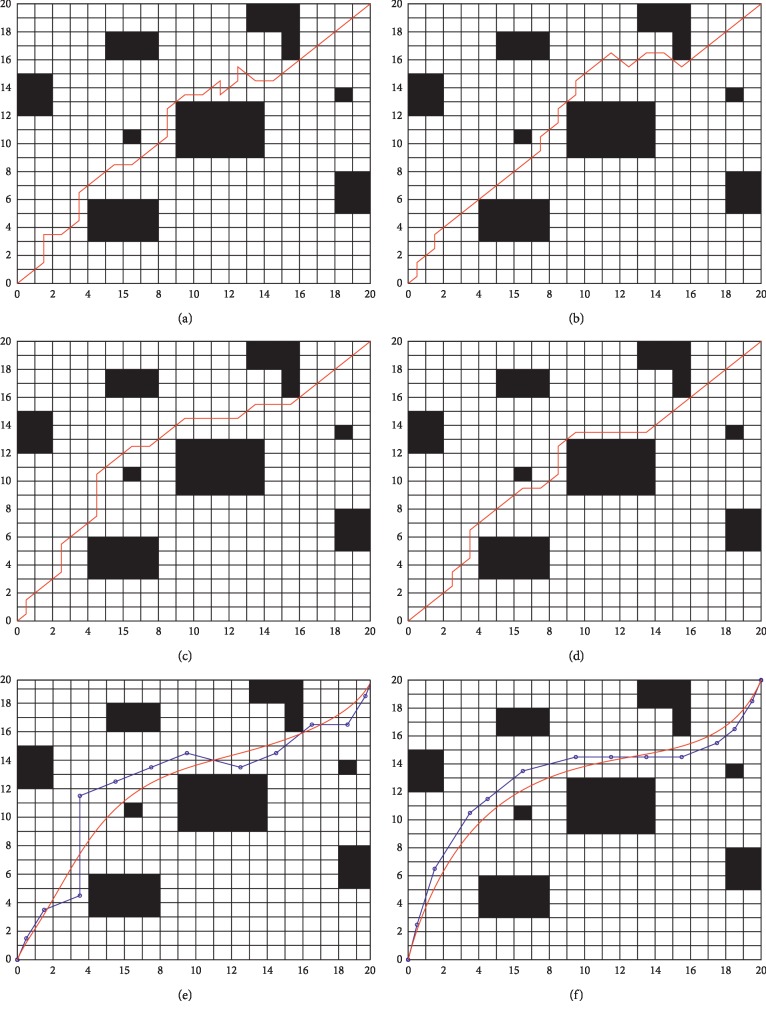
Path planning simulation results in environment 1. (a) Path planned by ACO; (b) path planned by the GA; (c) path planned by the ACO-GA; (d) path planned by the ASFA-GA; (e) path planned by the BCA; (f) path planned by the BCA-Q.

In the experiments, the traditional ant colony optimization (ACO), the traditional genetic algorithm (GA), ant colony optimization combined with the genetic algorithm (ACO-GA), the artificial fish swarm algorithm (ASFA-GA), the Bezier curve smoothing algorithm (BCA), and the Bezier smoothing algorithm with increased safety distance (BCA-Q) are adopted to conduct experiments in environment 1.  Figure 4 shows the path planning results with different algorithms. In order to eliminate the influence of random and other contingency factors on the algorithm, the above algorithms are executed 30 times independently, and the statistical results are given in Table 1, where “—” means that statistical results cannot be obtained.

Combining Figure 4 and Table 1, the following can be found in terms of path length. (1) ACO, the GA, and the ASFA-GA can plan a collision-free path (as shown in Figures 4(a), 4(b), and 4(d)) with the path lengths being 34.6248, 32.8663, and 31.2144, respectively. Compared with the BCA (29.9416), the planning paths of ACO, the GA, and the ASFA-GA are longer, which is caused by a lot of redundant nodes and redundant infection points in the path. (2) When the GA is introduced into ACO, i.e., for the ACO-GA, the path length is 31.7964; this is an improved performance compared with just ACO and just the GA. However, there are still peak inflection points (e.g., Figure 4(c)). (3) It can be seen from Figures 4(e) and 4(f) that the quality of the path obtained by the BCA and BCA-Q has been significantly improved; here, the blue circles are the control points of the Bezier curve, and the red line is the optimal smooth path. The path lengths are 29.9416 and 31.1843, respectively. Combined with Table 1, the minimum and average values of the planned path lengths obtained by using the BCA and BCA-Q are better than those of other methods. The reason for this is that the improved algorithm reduces redundant inflection points and redundant nodes in the path planning; therefore, the path is smoother and shorter. (4) Although the path length planned in Figure 4(f) is longer than that shown in Figure 4(e), the mobile security of the robot is guaranteed, and the path planning performance is better than that of other algorithms.

In terms of running time, Table 1 shows that the ASFA-GA is the best algorithm in terms of required time for simulations. Moreover, the ACO-GA has increased simulation time due to the introduction of the GA. This is because the GA introduces crossover and mutation operations in the process of path planning. Finally, Table 1 shows that although the simulation times of the BCA and BCA-Q are longer than those of other algorithms, their minimum path lengths are shorter. Therefore, compared with the traditional algorithms, the proposed algorithm has better performance in terms of path length and smoothness in the environment with low real-time requirements.

### 4.2. Effectiveness Experiment of Bezier Smoothing Algorithms

**Figure 5:**
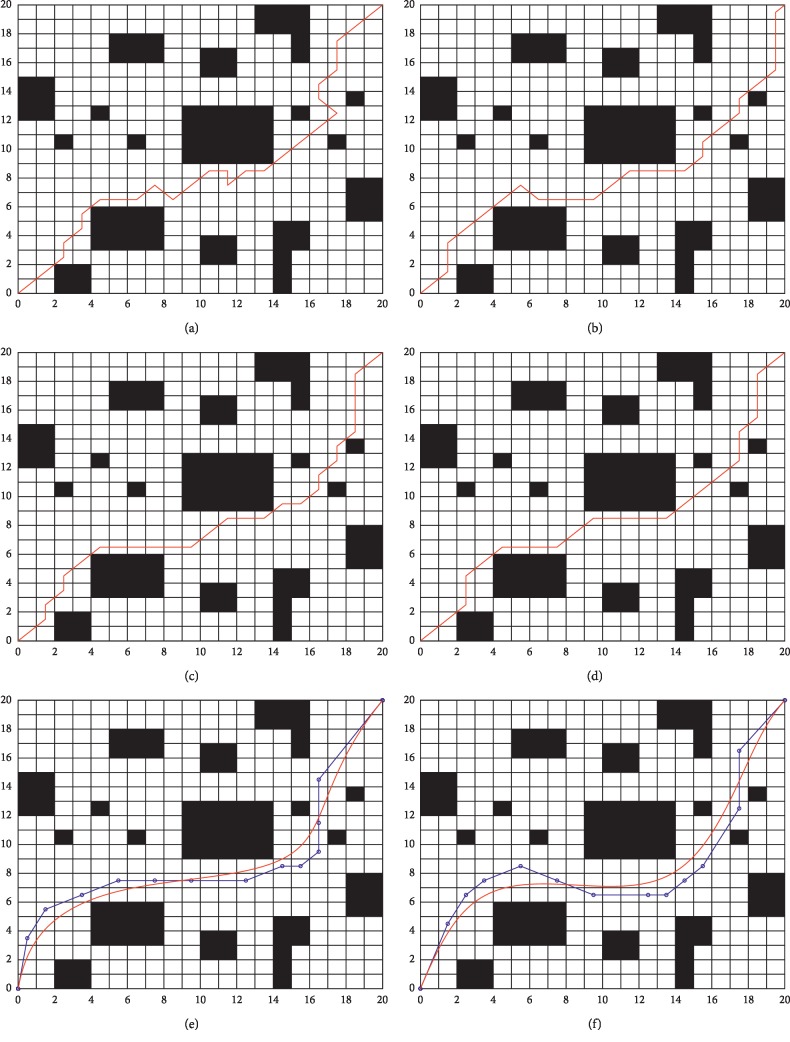
Path planning simulation results in environment 2. (a) Path planned by ACO; (b) path planned by the GA; (c) path planned by the ACO-GA; (d) path planned by the ASFA-GA; (e) path planned by the BCA; (f) path planned by the BCA-Q.

In order to verify the performance of the proposed algorithm, a complex grid environment of 20 × 20 is established in this paper. The algorithms shown in Section 4.1 are applied in environment 2.  Figure 5 shows the path planning results with different algorithms. In order to eliminate the influence of random and other contingency factors on the algorithm, the aforementioned algorithms are executed 30 times independently, and the statistical results are recorded in Table 2, where “—” means that statistical results cannot be obtained.

Based on the analysis of Figure 5 and Table 2, it can be observed that the obstacle coverage of environment 2 is increased by 5.75% compared with environment 1. However, the added coverage makes it more difficult to search for the global optimal solution. Compared with Table 1, the simulation time and distance of path planning in Table 2 are increased. The path length of the ASFA-GA is 32.3821. Although the path is not the longest of all algorithms, there are obvious inflection points (see Figure 5(d)). (2) From Figures 5(a)–5(d) and Table 2, one can see that ACO and the ACO-GA can also find a collision-free path in complex environments, but their average path length is longer compared to the ASFA-GA, which is 33.6631. We can also observe from Figure 5(e) that the path obtained by the BCA is smoother and shorter compared with those obtained by other algorithms. Redundant nodes are avoided, and the path length is 30.3458. From Table 2, we can see that the BCA is slightly insufficient compared to other algorithms in terms of simulation time consumption. However, it is still effective in complex environments from the perspective of path length and smoothness. Additionally, although the inflection point disappears in the path planned by the BCA, the path is very close to the obstacle. By increasing the safety distance in the fitness function, the BCA-Q improves the security of the mobile robot, which can be verified in Figure 5(f). However, this advantage is achieved by sacrificing the path length. The planned path length of the BCA-Q is 31.8503, which is 4.96% greater than that of the BCA. It is noteworthy that the path length of BCA-Q is still shorter than those of ACO and the GA. Moreover, its smoothness and path security are greatly improved.

## 5. Conclusions

In this paper, a new smooth path planning method combining genetic operators and Bezier curves has been proposed for mobile robots. First, the control points of the Bezier curve were determined by a genetic operation, and the smoothing characteristics of the Bezier curve were used to make the planning path smoother and more consistent, which reduced the energy loss in robot movement. Second, the safety distance was added to the fitness function and could be dynamically adjusted according to the distance between the path and the obstacle to ensure the safe and efficient movement of the robot. Finally, the simulation results showed that the proposed algorithm was effective in finding an optimal path; this optimal path was shorter, smoother, and safer than those obtained by traditional algorithms. However, the simulation time was increased. This was because the improved algorithm paid more attention to the quality of the solution in the process of path planning. There still exist various meaningful topics to be addressed, such as how to select the most appropriate number of control points of the continuous Bezier curve, how to apply the algorithm to more complex practical environments (such as rooms or other special environments), and how to improve the calculation efficiency with more control points.

## Figures and Tables

**Table 1:** Path planning statistics in environment 1.

Path planning algorithm	Planning path length			Simulation time consumption (s)		
	Minimum	Maximum	Average	Minimum	Maximum	Average
ACO	34.6248	50.3794	42.5416	10.4718	20.4864	15.6471
GA	32.8663	38.6874	35.7144	50.4718	70.1453	61.4772
ACO-GA	31.7964	35.4147	33.4172	59.5674	84.4792	67.3358
ASFA-GA	31.2144	33.5479	32.3648	8.4716	—	—
BCA	29.9416	32.4471	30.9436	136.4794	264.1479	202.4861
BCA-Q	31.1843	34.8517	32.9973	174.6659	296.3371	234.4781

**Table 2:** Path planning statistics in environment 2.

Path planning algorithm	Planning path length			Simulation time consumption (s)		
	Minimum	Maximum	Average	Minimum	Maximum	Average
ACO	35.4526	56.1147	45.9981	25.7242	38.7413	32.5469
GA	33.7963	40.4483	37.5448	85.7157	107.6814	96.1479
ACO-GA	32.9688	37.3691	35.1472	108.4743	136.4731	121.1173
ASFA-GA	32.3821	34.7728	33.6631	20.4473	—	—
BCA	30.3458	33.7694	32.1473	202.7781	378.9283	293.4417
BCA-Q	31.8503	35.4473	33.7628	286.5575	402.6491	345.4799

## Data Availability

The data used to support the findings of this study are included within the article. All required models and parameters are listed in the article.
